# Identification and Nutrient Composition of a Wild *Pleurotus pulmonarius* Strain from Tibet, and the Antioxidant and Cytotoxic Activities of Polysaccharides from This Fungus

**DOI:** 10.3390/foods14071198

**Published:** 2025-03-28

**Authors:** Hao Jiang, Lei Gao, Xin Hu, Junsheng Fu, Junli Zhang

**Affiliations:** 1 College of Life Sciences, Fujian Agriculture and Forestry University, Fuzhou 350002, China; jianghaohh23@163.com (H.J.); hx1962024@163.com (X.H.); fujunsheng81@163.com (J.F.); 2 Tibet Academy of Agricultural and Animal Husbandry Sciences, Lhasa 850000, China; gaoleilei3@163.com

**Keywords:** *Pleurotus pulmonarius*, fungi identification, nutrient composition, antioxidant activity, cytotoxicity

## Abstract

The selection and breeding of high-quality wild edible fungal strains can bring significant economic and social benefits. A wild fungal strain (X21185) from the Tibetan Plateau was isolated and identified as a novel *Pleurotus pulmonarius* (*P. pulmonarius*) based on its morphological and molecular characteristics. The appropriate culture conditions for *P. pulmonarius* were determined. The nutrient contents of *P. pulmonarius* fruiting bodies were analyzed. Compared with the conventional nutritional contents of the representative edible fungi (*Pleurotus ostreatu* and *Pleurotus eryngii*) and egg, the protein, ash, and dietary fiber contents of *P. pulmonarius* were higher. Four types of essential amino acids, seven types of nonessential amino acids, the total essential and nonessential amino acids of *P. pulmonarius* were present in considerably higher quantities than those of representative edible fungi (*Pleurotus ostreatus* and *Pleurotus citrinopileatus*) and egg, respectively. *P. pulmonarius* polysaccharides (PPPs) had strong ABTS^+^, DPPH, and hydroxyl free radical scavenging activities (EC_50_: 0.051, 3.322, and 2.87 mg/mL, respectively), and the cytotoxicity was higher against HepG2 hepatocellular carcinoma cells (IC_50_: 1.501 mg/mL) than against MDA-MB-468 triple-negative breast cancer cells (IC_50_: 2.183 mg/mL). This study provides a foundation for the development of the novel wild *P. pulmonarius* strain.

## 1. Introduction

Edible fungi are macrofungi that mainly belong to the phylum Basidiomycota; they have been used as a dietary food source for thousands of years [1], they are key sources of amino acids, proteins, carbohydrates, fats, vitamins, and minerals, and they have been developed as nutraceuticals and cosmeceuticals [2,3,4]. At present, China and other Asian and European countries not only have rich resources of edible fungi, but also have thousands of years of medicinal history. According to the statistics of the China Edible Fungus Association, the production of edible fungus in China reached 39.6 million tons in 2019, with an output value of more than USD 43 billion [5]. The fruit bodies of edible fungi are commonly referred to as mushrooms. Polysaccharides isolated from these edible fungi have shown a wide range of biological activities, such as antioxidant, antitumor, and many other activities [6]. Oyster mushrooms have been recognized as mushrooms with dual functions for humans, both as food and medicine, and they currently rank as the second most vital mushrooms for trade and the third most commercially produced mushrooms globally among edible fungi. Among the various species of oyster mushrooms, *Pleurotus ostreatus* is the most extensively cultivated, followed by *Pleurotus pulmonarius* (*P. pulmonarius*) [7]. *P. pulmonarius* belongs to the division Basidiomycota, class Agaricomycetes, order Agaricales, family Pleurotaceae, and genus *Pleurotus. P. pulmonarius* is a well-known edible mushroom with high nutrient contents, has become increasingly popular in Asian countries, and has significant medicinal values. Its therapeutic properties encompass anticancer effects, antioxidant, hypocholesterolemic, and anti-atherogenic capabilities, etc. [8]; it stands out among the extensively cultivated edible mushrooms, and is widely used in the food, pharmaceutical, and biotechnology industries [8,9]. It was reported that *P. pulmonarius* possess antioxidative, anti-inflammatory, antimicrobial, enzyme-inhibitory, and anticancer activities [10,11,12,13,14]. The bioactive and antioxidant components of *P. pulmonarius* mainly include high-weight compounds (polysaccharides, etc.) and low-molecular-weight compounds (polyphenols, etc.) [7,15]. The contents, chemical profiles, and many biological activities of low-molecular-weight compounds have been reported [7,15]. However, the antioxidant activity of *P. pulmonarius* polysaccharides (PPPs) is still unclear.

In humans, excessive reactive oxygen species (ROS) accelerate aging and cause oxidative damage, cancer, and many other diseases [15]. Cancer remains the second most common cause of death after heart disease according to the 2023 Cancer Statistics Report of the United States. Many mushroom polysaccharides that are cytotoxic to tumor cells can be developed into anti-tumor products [16]. Liver cancer or hepatocellular carcinoma is the fifth most prevalent cancer and the third leading cause of all cancer-related deaths in the world [13]. Breast cancer prevalence has increased to 43.5 million, including over 18 million new cases and 9.6 million mortalities [17]. Existing chemotherapy involves many anticancer drugs with numerous side effects. Therefore, cancer therapy may require the use of additional adjuvants to improve and save patient status. As good adjuvants, mushroom agents that have antiproliferative features with minimal side effects can provide an increasing rate of curability for cancer patients [17]. The polysaccharides and other macromolecules in mushrooms of the genus *Pleurotus* have been shown to possess various therapeutic effects such as antioxidant, anticancer (antihepatocellular carcinoma, antibreast cancer, etc.), and many other activities; furthermore, the polysaccharide–protein complex isolated from *P. pulmonarius* could suppresses liver cancer development and progression, and PPP could suppress human colon adenocarcinoma cell line proliferation and glioma growth [13,14,18,19]. However, the inhibitory effects of *P. pulmonarius* on liver cancer and breast cancer are still unclear.

In recent years, due to excessive harvesting and habitat destruction, *P. pulmonarius* wild resources have plummeted. Although *P. pulmonarius* can be cultivated artificially, its mycelial growth rate is slow and the yield is low; selecting and breeding wild *P. pulmonarius* strains of high quality will greatly promote the artificial industrial cultivation of *P. pulmonarius*.

Fungi produce diverse primary and secondary metabolites depending on environmental conditions and other external factors [20]. Tibet is located in high-altitude southwest China; the mean altitude is above 4000 m, and the special geographical location and the complex and varied climate of Tibet nurture a great variety of mycorrhizal resources, so it is a unique edible fungus production area [16,20]. For example, renowned *Ophiocordyceps sinensis* sourced from Tibet shows superior quality and efficacy compared to that obtained from other areas [20]. It is of immense social and economic value to introduce excellent and novel edible fungi from Tibet to low-elevation areas for domestication and popularization. A wild *Pleurotus citrinopileatus* found in Tibet was successfully domesticated and cultivated in low-altitude areas, and the polysaccharides of *Pleurotus citrinopileatus* were proved to have strong antioxidant and anticancer effects [16]. Fortunately, after extensive exploration, we have also discovered a novel wild strain (X21185) of *P. pulmonarius* in the Tibet Plateau. The appearance of this wild strain is white, different from the traditional non-white appearance of *P. pulmonarius*. Its impressive environmental adaptability and stress resistance in the unique environmental conditions of the Tibet Plateau and its performance and advantages need to be explored. Therefore, the following contents were investigated in this study: the wild strain (X21185) of *P. pulmonarius* was isolated and identified according to morphological and molecular characteristics; its biological characteristics of mycelial growth were determined; the fruiting bodies of *P. pulmonarius* were cultivated; the nutrients of *P. pulmonarius* fruiting bodies were analyzed; and the antioxidant activities and cytotoxicities of PPP on hepatoma cells (HepG2) and triple-negative breast cancer cells (MDA-MB-468 cells) were determined. These results provide a scientific basis for the full development and utilization of *P. pulmonarius*.

## 2. Materials and Methods

### 2.1. Sample Collection and Preparation

Wild mycorrhizal fungal substrate (strain no. X21185) was collected from Yigong Township, Bomi County, Tibet Autonomous Region of China. The strain was isolated from the fruiting bodies, and the internal transcribed spacer (ITS) regions of this strain were analyzed to determine species identification. Small pieces were plated on potato dextrose agar (PDA) and incubated at 25 °C for 5 days [21]. The purified cultures were maintained on several PDA slants.

### 2.2. Isolation and Identification

The wild strain was identified based on the following morphological and molecular characteristics.

#### 2.2.1. Morphological Identification

For morphological identification, observations of this strain were compared to those by Huang Nian Lai and Wei Jing Chao [22,23].

#### 2.2.2. Molecular Identification

DNA was extracted from the mycelium of this wild strain using a Fungal DNA Extraction Kit (OMEGA, Fuzhou Nanjiang Biotechnology Co., Ltd., Fuzhou, China) in accordance with the manufacturer’s instructions. PCR amplification of the ITS sequences was conducted using ITS1 (5′-TCCGTAGGTGAACCTGCGG-3′) and ITS4 (5′-TCCTCCGCTTATTGATATATGC-3′) primers and the following procedure [24]: the samples were denatured at 95 °C for 5 min, followed by 35 cycles of denaturing at 95 °C for 45 s, annealing at 58 °C for 50 s, and extension at 72 °C for 45 s; the samples were then extended at 72 °C for 10 min; and next, 3 μL PCR products were separated by 1% agarose gel electrophoresis. Purified PCR products were sent to Fuzhou Moby Dick Bio technology Co., (Fuzhou, China) for sequencing. The obtained ITS sequences were inputted into the NCBI Nucleotide Database (http://www.ncbi.nlm.nih.gov, accessed on 11 March 2024) for blast comparison. The ITS sequences with a high degree of similarity were downloaded. A phylogenetic tree was then constructed using the maximum-likelihood method.

### 2.3. Biological Characteristics of Mycelial Growth

Mycelial growth characteristics were evaluated based on the method of Cao et al. [25], with slight adjustments. Briefly, the strain was cultured on PDA medium for 7 days, inoculated in corresponding Petri dishes, and then cultured at 25 °C in the dark.

(1) $$\mathrm{Mycelial}\;\mathrm{growth}\;\mathrm{rate}\;(\mathrm{mm}/d)=\frac{colony\;diameter\;(mm)}{number\;of\;days\;of\;growth\;(d)}$$

#### 2.3.1. Carbon Source

To assess the effects of the carbon source, carbon source exploration media consisting of 200 g peeled potato, 20 g carbon sources (glucose, sucrose, fructose, maltose, mannose, or starch), 5 g peptone, 1.5 g MgSO_4_, 2 g K_2_HPO_4_, 10 mg vitamin B1, 20 g agar, and 1 L water (neutral pH) were used.

#### 2.3.2. Nitrogen Source

To assess the effects of the nitrogen source, nitrogen source exploration media consisting of 200 g peeled potato, 20 g glucose, 5 g nitrogen sources (urea, yeast powder, ammonium sulfate, beef powder, peptone, or ammonium nitrate), 1.5 g MgSO_4_, 2 g K_2_HPO_4_, 10 mg vitamin B1, 20 g agar, and 1 L water (neutral pH) were used.

#### 2.3.3. Temperature

To assess the effects of temperature, a constant-temperature incubator at 15, 20, 25, 30, 35, and 40 °C in the dark was used.

#### 2.3.4. pH

To assess the effects of pH, 1.0 mol/L NaOH and 1.0 mol/L HCl solutions were added into the media to alter the pH to 5.0, 6.0, 7.0, 8.0, 9.0, or 10.0.

### 2.4. Cultivation of P. pulmonarius Fruiting Bodies

The materials for cultivation (comprising 60% wood chips, 20% cottonseed hulls, 18% bran, 1% lime, and 1% sugar, based on mass fractions) were thoroughly mixed with water (60% moisture content), segmented into bags for cultivation, sterilized at 121 °C for 3 h, cooled, inoculated with *P. pulmonarius* (X21185) in an ultraclean workbench, and then transferred to a culture chamber. Cultivation of the fungus occurred in darkness at temperatures ranging from 22 to 25 °C [16]. The mycelium was monitored, including for contamination. When each bag was full of mycelium, it was transferred to a mushroom chamber and the environmental factors (light, temperature, and humidity) were adjusted to achieve optimum growth. The growth and maturation processes of the protomatrix and stroma were observed and recorded [16].

### 2.5. Nutritional Analysis of Fruiting Bodies

The fresh fruiting bodies of *P. pulmonarius* were dried to a constant weight at 50 °C and kept sealed until use. The nutritional values of the fruiting bodies were determined according to the methods of the Association of Official Analytical Chemists [16]. Moisture content was determined using the direct drying method, total nitrogen content was estimated using the Kjeldahl method, and total protein was then calculated, ash content was determined using the high-temperature burning method, dietary fiber content was determined using the enzyme weight method, sodium content was determined using flame atomic emission spectrometry, lipids were extracted in a Soxhlet apparatus using petroleum ether, and lipid content was determined using gravimetric analysis.

Amino acid contents were determined using an automatic amino acid analyzer (L-8900, Hitachi, Tokyo, Japan). Briefly, 0.1 g of mushroom powders was hydrolyzed in a threaded test tube with 50 mL of 6 mol/L HCl and 5 mg/mL phenol at 110 °C for 22 h. The hydrolysates were filtered into a 200 mL volumetric flask and diluted with water. A total of 1.5 mL of the hydrolysate was dried at 60 °C and then redissolved in 1.5 mL water, and this drying and dissolving process was repeated three times. Finally, the dried samples were dissolved in 2 mL of 0.02 mol/L HCl and filtered through a 0.22 µm filter membrane for analysis [16].

### 2.6. Antioxidant Activity of PPP

#### 2.6.1. Preparation and Quantification of PPP

Water-soluble polysaccharides were extracted from *P. pulmonarius* fruiting bodies. Briefly, pretreated dried samples were subjected to extraction in water at 80 °C three times (30:1 mL/g of liquid/solid ratio). The aqueous extracts were concentrated to 20% of the original volume under reduced pressure in a rotary evaporator. Proteins were then removed using the Sevag method [26]. To obtain the crude polysaccharides, the extracts were precipitated with three times their volume of 95% ethanol at 4 °C overnight and then centrifuged at 8400× *g* for 15 min. Lastly, the precipitates were dissolved in distilled water and freeze-dried.

The contents of these crude polysaccharides were measured according to the phenol-sulfuric acid colorimetric method, with D-glucose as a standard at 490 nm [27]. The sugar solution (0.6 mL) and the phenol solution (0.3 mL) were added into screw-cap tubes (13 × 100 mm), which were capped and vortex-stirred. Then, 1.5 mL of concentrated sulfuric acid was added slowly down the side of the tubes. These tubes were then closed, vortex-stirred for 5 s, and then incubated for 15 min at 100 °C [27]. All tubes were allowed to cool down to room temperature before reading the absorbances at 490 nm using distilled water as blank in a spectrophotometer (SP-756P, Shanghai Spectrum Instruments, Shanghai, China). The yield of polysaccharides was found to be 90.6% based on the equation fitting the standard curve for glucose:

$$y=0.4968x-0.2818,R^{2}=0.9913$$

where y and x represent the values of absorbance and the concentrations of glucose solution, respectively.

#### 2.6.2. ABTS Free Radical Scavenging Activity

ABTS free radical scavenging activity was assessed according to the method described by Miller et al. [28], with slight adjustments. Briefly, PPPs in water (0.025, 0.05, 0.25, 0.5, 1.2, or 5 mg/mL) were mixed with ABTS^+^ solution (diluted with water to an absorbance at 734 nm of 0.7 ± 0.02) and incubated at room temperature for 20 min, and the absorbances at 734 nm were assessed. The rates of ABTS radical scavenging activity were calculated using the following equation [28]:

(2) $$Scavenging\;activity\left(\%\right)=(1-\frac{Asample}{Acontrol})\;\times \;100$$

where A_sample_ and A_control_ represent the absorbances of ABTS^+^ solutions with and without PPP, respectively.

#### 2.6.3. DPPH Radical Scavenging Activity

DPPH radical scavenging activity was assessed according to the method described by Blois et al. [29], with slight adjustments. Briefly, 100 μL PPP (0.025, 0.05, 0.25, 0.5, 1.2, or 5 mg/mL) was mixed with 400 μL methanol containing 0.1 μL DPPH solution, incubated in the dark for 20 min, and then the absorbances at 517 nm were assessed, and 500 μL DPPH solution was used as the blank control. The rates of DPPH radical scavenging were calculated using the following equation [29]:

(3) $$Scavenging\;activity\left(\%\right)=(1-\frac{Asample}{Acontrol})\;\times \;100$$

where A_sample_ and A_control_ represent the absorbances of DPPH solutions with and without PPP, respectively.

#### 2.6.4. Hydroxyl Radical Scavenging Activity

Hydroxyl radical scavenging activity was assessed according to the method described by Hifney et al. [30], with slight adjustments. The assessment involved hydroxyl free radicals of 9 mM FeSO_4_ and 1 mL of 0.3% H_2_O_2_ in 0.5 mL of 9 mM salicylic acid–ethanol solution, made up to 5 mL using distilled water, and then incubated at 37 °C for 30 min. The absorbances at 510 nm were assessed. The rates of hydroxyl radical scavenging activity were calculated using the following equation [30]:

(4) $$Scavenging\;activity\left(\%\right)=(1-\frac{Asample}{Acontrol})\;\times \;100$$

where A_sample_ and A_control_ represent the absorbances of sample solutions with and without PPP, respectively.

#### 2.6.5. Ferric Ion Reducing Antioxidant Power (FRAP)

The evaluation of FRAP was assessed according to Xiao et al. [16], with slight adjustments. Three milliliters of FRAP working solution was mixed with 1.5 µL sample solution and reacted for 15 min at 37 °C, and OD_593_ was recorded. Distilled water was used to replace the polysaccharide as the blank. The FRAP working solution was prepared using 0.3 mol/L acetic acid–sodium acetate buffer solution (pH 3.6), 0.02 mol/L FeCl_3_ solution, and 0.01 mol/L TPTZ (2,4,6-tris(2-pyridyl)-1,3,5-triazine) solution in a volume ratio of 10:1:1. To create a standard curve, 0.5 mL of 0.025–1.5 mmol/L FeSO_4_ solutions was mixed with 3.0 mL FRAP working solution, reacted at 37 °C for 15 min, and then subjected to OD_593_ measurement to construct the standard curve. For the control, distilled water was used to replace the FRAP working solution. FRAP values were determined based on the differences between OD_593_ measurements and the corresponding FeSO_4_ concentration on the standard curve [16].

### 2.7. MTT Assay of Cytotoxicity

The cytotoxic effects of PPP on hepatoma cells (HepG2) and triple-negative breast cancer cells (MDA-MB468 cells) were evaluated by using 3-(4,5-dimethylthiazol-2-yl)-2,5-diphenyl tetrazolium bromide (MTT) assays; these were performed using the method of Suresh [31]. To investigate the proliferation of the cancer cells in response to the PPP, the cells were cultured in fresh DMEM/F12 complete medium. The primary cells were seeded into 96-well microplates. After 12 h, the cells were treated with PPP at different concentrations (0, 0.05, 0.25, 1, 2, and 5 mg/mL, respectively) for 24 h in a 5% CO_2_ incubator at 37 °C without light. After adding 50 μL MTT solution (5 mg/mL) into each well of the microplate, the cells were incubated in the 5% CO_2_ incubator at 37 °C without light for 4 h. Then, 150 μL of DMSO was added to dissolve the formazan crystals. The absorbances were measured using an enzyme-linked immunosorbent assay (ELISA) plate reader at 490 nm after 1 h. The impacts of varying PPP levels on the half-maximal inhibitory concentration (IC_50_) were ascertained by evaluating the cell survival rates through the following equation [31]:

(5) $$Cell\;survival\left(\%\right)=\frac{Asample-Ablank}{Acontrol-Ablank}\times 100$$

where Asample and Ablank represent the absorbances of sample solutions with and without PPP, respectively.

### 2.8. Statistical Analysis

The data were analyzed according to one-way analysis of variance (ANOVA) followed by Duncan’s multiple range test. The data were presented as mean ± SD, and *p* ≤ 0.05 was considered statistically significant.

## 3. Results and Discussion

### 3.1. Identification of Wild P. pulmonarius from Tibet

The wild strain was identified based on both morphological and molecular characteristics.

#### 3.1.1. Morphological Characterization of Fruiting Bodies

**Figure 1:**
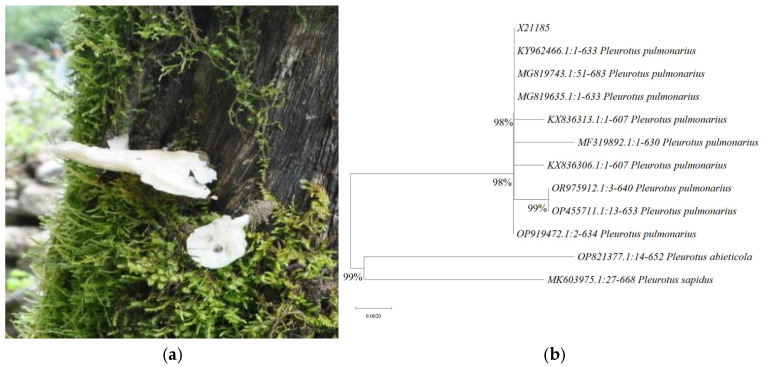
Morphological and molecular identification of wild *P. pulmonarius* (X21185). Wild *P. pulmonarius* fruit body found in Tibet Plateau (**a**); maximum likelihood phylogenetic tree based on ITS sequences (**b**).

Both the wild and the cultivated fruiting bodies were observed. The pileus was semi-circular, scalloped, kidney-shaped, shell-shaped, or round. The edges of the lamellae initially curled inward, but later flattened out. The center was slightly depressed or had a slightly stipe appearance, was white, and had a smooth surface. The gills were somewhat dense, short, extended, and varied in length. The stem was short or absent, and often found on the side. The flesh was tough and white to milky white (Figure 1a). The appearance of traditional *P. pulmonarius* is generally displayed as a non-white color. Therefore, this wild strain (X21185) was preliminarily identified as a novel *P. pulmonarius* species according to the color of appearance.

#### 3.1.2. Molecular Characterization of Fruiting Bodies

The traditional morphological identification methods for edible fungi are not sufficiently accurate. The endogenous reference gene detection method is a recently developed and reliable method for the identification of edible fungi [32]. ITS identification refers to a method of DNA sequencing the ITS sequence and comparing the sequenced ITS sequence with known fungal ITS sequences to obtain information on the species and genus of the unknown fungi. The amplified ITS sequences can then be sequenced, and the resulting sequences can be analyzed using software to compare them with known fungal ITS sequences [10,33]. This comparison allows for the identification of the species and genus of the unknown fungi. The maximum likelihood method was used to construct a phylogenetic tree of ITS sequences of *P. pulmonarius*. The PCR products of the wild strain (X21185) were 634 bp in length, and the ITS sequences of this strain had 98% similarity with the reported sequences of these *P. pulmonarius* strains, and were genetically distant from other *Pleurotus* spp. (*Pleurotus abieticola* and *Pleurotus sapidus*) (Figure 1b). Therefore, this wild strain (X21185) was identified as a novel *P. pulmonarius* species according to the morphological observation and endogenous reference gene detection methods. Two species of *P. pulmonarius* were identified based on the morphological features and DNA sequence information [10,33].

### 3.2. Biological Characteristics of Mycelial Growth

**Figure 2:**
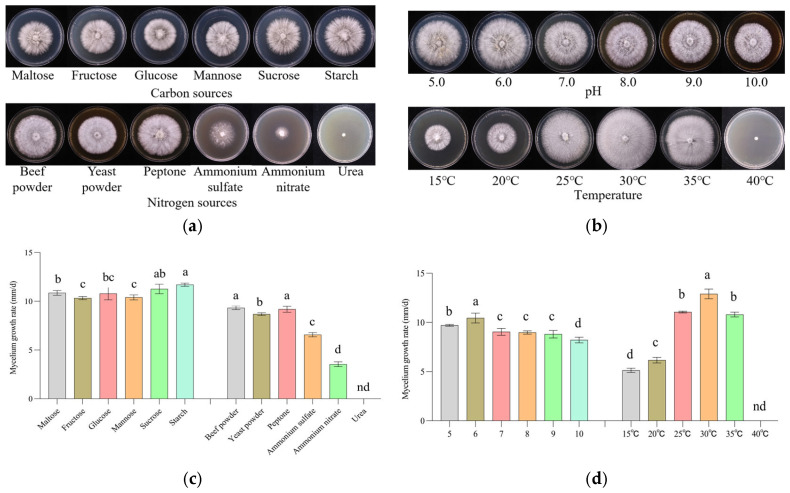
*P. pulmonarius* (X21185) mycelial growth. (**a**) Growth trend diagrams of carbon and nitrogen sources. (**b**) Growth trend diagrams of pH and temperature. (**c**) Mycelial growth rate bar charts of carbon and nitrogen sources. (**d**) Mycelial growth rate bar charts of pH and temperature. In the bar charts (**c**,**d**), the same letters indicate no significant difference, while different letters indicate a significant difference (*p* < 0.05), nd, not detected.

The carbon source directly affects mycelial growth and yield [34]. The strain grew well in all kinds of carbon sources. The mycelial growth rate was optimum in the starch group (11.70 mm/d), slightly higher than that of the sucrose group and significantly higher than those of the other carbon source groups (*p* < 0.05), with dense and robust mycelium exhibiting regular edges. Based on the mycelial growth rate and growth potential, starch was the optimum carbon source (Figure 2a,c). The nitrogen source is also an important factor affecting microbial growth and metabolism [35]. Beef powder significantly promoted mycelial growth, while ammonium nitrate and urea hardly promoted mycelial growth. The mycelial growth rate was optimum in the beef powder group (9.33 mm/d), slightly higher than that of the peptone group, but significantly higher than those of the other groups (*p* < 0.05) (Figure 2a,c). During edible fungi cultivation, pH regulation is also a key factor to improve the yield and quality [16]. The mycelial growth rate was optimum (10.46 mm/d), and the mycelial growth rate was the fastest when pH value was at pH 6; the branches were more developed, and the nutrients in the culture medium were utilized more efficiently (Figure 2b,d). Temperature is a key factor affecting the mycelial growth of edible fungi [36]. The aerial mycelial growth was the most vigorous and the mycelial growth rate was optimal at 30 °C (12.90 mm/d), clearly different from those at other temperatures. The mycelium stopped growing at 40 °C (Figure 2b,d).

Combining the results of the above experiments, it was concluded that the appropriate culture conditions for *P. pulmonarius* (X21185) were as follows: starch was used as carbon source, beef powder was used as nitrogen source, pH value was pH 6, and the temperature was 30 °C (Figure 2). These findings provide the possibility for adapting and scaling up the cultivation of *P. pulmonarius* from high-altitude Tibet to low-altitude regions.

### 3.3. Cultivation of P. pulmonarius Fruiting Bodies

**Figure 3:**
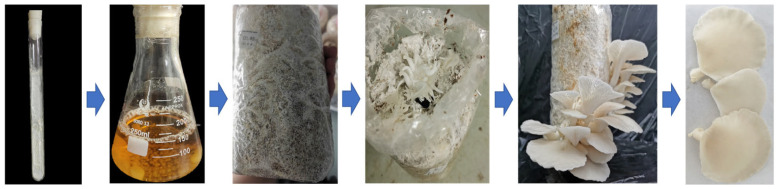
Domestication and cultivation process.

The wild *P. pulmonarius* (X21185) strain was domesticated in cultivation bags under optimum mycelial cultivation conditions for 25 days. During the early stage of fruiting body production (about 7 days), white primordia appeared on the culture surface. After the middle stage of differentiation, the light white structures with slightly curled edges expanded. After maturity, the structures with light white caps were medium-sized. The mean fresh weight of a mature harvested fruiting body was 45.33 g (Figure 3). The results showed that *P. pulmonarius* (X21185) had a short growth cycle and high yield.

### 3.4. Nutrient Contents of P. pulmonarius Fruiting Bodies

#### 3.4.1. Conventional Nutrient Contents

Conventional nutrient analysis of the cultivated *P. pulmonarius* fruiting bodies showed that the protein content (26.3%) was higher than the nutritional standard for edible mushrooms (24.00%) and that of *Pleurotus ostreatus* (17.06%, the most extensively cultivated oyster mushroom) [37,38]. The total sugar content was 7.10%, which was more than ten times higher than that of egg (0.70%, a well-known and widely accepted nutrient-rich food), and was more than those of other important edible mushrooms—*Pleurotus eryngii* (6.85%, a very important edible fungi) and *Pleurotus citrinopileatus* (4.50%, one of the most extensively cultivated edible fungi) [16,39,40]. The fat content was 1.30%, which was much lower than that of egg (8.60%), and higher than that of *Pleurotus eryngii* (0.56%) and *Pleurotus ostreatus* (1.21%) [39,40]. The dietary fiber content (which was not present in egg) was 41.80% (Table 1), which was higher than those of *Pleurotus ostreatus*, *Pleurotus citrinopileatus*, and *Pleurotus eryngii*. Even the content of Na reached 16.10 mg/100 g. Therefore, based on these data, *P. pulmonarius* is a type of tasty, high-protein, high-fiber, and low-fat food. It is expected to become a major foodstuff in addition to beans, cereals, and other plant-based foodstuffs that are high in proteins [41].

**Table 1:** Nutrient contents of *P. pulmonarius* fruiting bodies.

Nutrient Composition	Content (g/100 g)				
	*P. pulmonarius*	*Pleurotus ostreatus* [38]	*Pleurotus eryngii* [39]	*Pleurotus citrinopileatus* [16]	Egg [40]
Moisture (%) (fresh)	94.40	91.01	-	91.70	75.20
Moisture (g) (dry)	9.50	6.46	9.16	11.40	-
Crude protein (g)	26.30	17.06	19.15	28.50	12.04
Total sugar (g)	7.10	-	6.85	4.50	0.70
Fat(g)	1.30	1.21	0.56	1.40	8.60
Dietary fiber (g)	41.80	23.63	27.50	34.00	0
Ash (g)	8.00	7.82	6.40	10.20	0.90
Na (mg)	16.10	-	-	13.00	131.50

-: Not detected.

#### 3.4.2. Amino Acid Composition

Amino acids are indispensable compounds in organisms, playing vital roles in a variety of biological activities [42]. Edible fungi are known for being a rich source of protein, with an amino acid composition comparable to animal and dairy products [43]. The *P. pulmonarius* fruiting bodies contained 15 types of amino acids, the most abundant of which was glutamate (a key amino acid in nitrogen metabolism in organisms and a major component of proteins) (Table 2). The total amino acid, essential amino acid, and nonessential amino acid contents were 19.20%, 5.86%, and 13.34%, higher than those of *Pleurotus ostreatus, Pleurotus citrinopileatus*, and egg, respectively [16]. Of the 15 types of amino acids identified, 4 types of essential amino acids and 7 types of nonessential amino acids with key nutritional values were present in considerably higher quantities than those of *Pleurotus ostreatus, Pleurotus citrinopileatus*, and egg, respectively [16,40]. In addition, the contents of glutamic acid (4.68%, an umami-flavored amino acid) and glycine (a sweet amino acid, 0.98%) were nearly triple and 2.5 times as high as that of egg (1.59 and 0.39, respectively), indicating potentially better taste and freshness compared to that of egg. The essential/total amino acid ratio was 0.30, and the essential/nonessential amino acid ratio was 0.44 (Table 2), which are close to the ideal values (0.40 and 0.60, respectively) proposed by the Food and Agriculture Organization/World Health Organization [16,44,45]. In conclusion, this wild strain is not only a high-protein edible mushroom, but also has more mineral elements than many other edible mushroom species, making it highly nutritious. It can be seen that the *Pleurotus pulmonarius* strain (X21185) is an ideal healthy ingredient. The results show that *P. pulmonarius* is a good source of carbohydrates, fibers, proteins, essential amino acids, and minerals.

**Table 2:** Amino acid contents of *P. pulmonarius* fruiting bodies (g/100 g).

Nutrient Composition		Contents (g/100 g)			
Amino Acid Composition		*P. pulmonarius*	*Pleurotus ostreatus* [38]	*Pleurotus citrinopileatus* [16]	Egg [45]
EAAs (essential amino acids)	Ile	0.57	0.53	0.49	0.65
	Val	0.98	0.83	0.93	0.64
	Lys	1.29	0.10	1.16	0.85
	Met	-	-	-	0.33
	Leu	1.17	1.23	1.02	1.05
	Phe	0.82	0.61	0.69	0.65
	Thr	1.03	0.78	0.93	0.59
	Trp	-	-	-	0.19
NEAA (nonessential amino acid)	Arg	1.33	0.11	0.89	0.74
	His	0.50	0.35	0.41	0.27
	Tyr	0.43	0.58	0.4	0.50
	Ala	1.24	0.14	1.14	0.66
	Pro	0.86	0.71	0.78	0.34
	Ser	1.15	0.75	1.08	0.91
	Glu	4.68	2.31	3.81	1.59
	Gly	0.98	0.87	0.99	0.39
	Asp	2.17	1.47	1.72	1.21
	Cys	-	-	-	0.50
EAAs (essential amino acids)		5.86	4.08	5.22	4.93
NEAA (nonessential amino acid)		13.34	7.29	11.18	7.11
TAA (total amino acid)		19.20	11.37	16.4	12.04
E/T (essential/total amino acid ratio)		0.30	0.35	0.32	0.41
E/N (essential/nonessential amino acid ratio)		0.44	0.56	0.44	0.69

-: Not detected.

### 3.5. Antioxidant Activities of PPP

Free radicals and oxidative stress can cause the disintegration of cell membranes and cell substances, which can damage living cells and lead to a variety of human diseases [46]. Due to the reactive oxygen species (ROS) scavenging activity, antioxidant molecules maintain cellular ROS homeostasis and thus prevent ROS toxicity. The endogenous and exogenous antioxidants scavenge ROS and help the human body to combat oxidative stress [15]. Polysaccharides from many edible fungi are considered as potential free radical scavengers (antioxidants) [47]. Therefore, the antioxidant activities of PPP were determined, and ascorbic acid was used as the positive control.

#### 3.5.1. ABTS Radical Scavenging Activity

**Figure 4:**
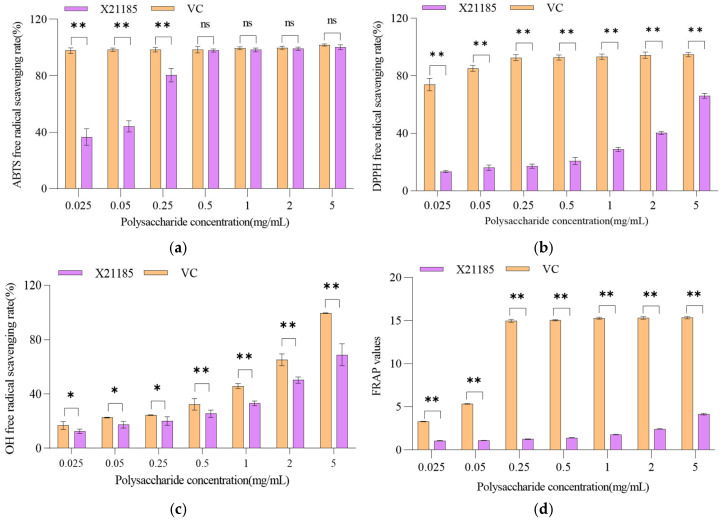
In vitro antioxidant activities of PPP. (**a**) ABTS free radical scavenging activity. (**b**) DPPH free radical scavenging activity. (**c**) Hydroxyl free radical scavenging activity. (**d**) Ferric ion reducing antioxidant power (FRAP). Ascorbic acid (vitamin C, VC) was the positive control. The data represent the mean ± SD of five independent experiments. * *p* < 0.05, ** *p* < 0.01, ns, non-significant.

Antioxidants react with and reduce ABTS^+^ (blue-green cation radical produced from ABTS by oxidation involving potassium persulfate), lightening its color. ABTS radical scavenging activity can be evaluated by measuring the change in the solution’s absorbances at a specific wavelength of 734 nm before and after the reaction [47]. The ABTS radical scavenging activity increased with the increases in PPP concentrations. When PPP concentration reached 0.50 mg/mL, the scavenging rate peaked at 97.87% (Figure 4a), which was much higher than that of *Pleurotus djamor* (a common oyster mushroom) polysaccharides (35.08%), and close to that of ascorbic acid (98.51%, nonsignificant) [48]. The half-maximal effective concentration (EC_50_) of PPP was 0.051 mg/mL, lower than that of *Pleurotus citrinopileatus* (0.063 mg/mL, Table 3), and this showed that the ABTS radical scavenging activity of PPP was stronger than that of *Pleurotus citrinopileatus*.

#### 3.5.2. DPPH Radical Scavenging Activity

The reduction of the free radical DPPH• to DPPH by the presence of a proton-donating substance is an indicator of antioxidant activity [49]. The DPPH radical scavenging activity increased as the concentrations of PPP increased. When PPP concentration reached 5.00 mg/mL PPP, the scavenging rate peaked at 66.07% (Figure 4b), which was lower than that of ascorbic acid; the EC_50_ was 3.322 mg/mL (Table 3).

#### 3.5.3. Hydroxyl Radical Scavenging Activity

Hydroxyl radicals are highly potent oxidant free radicals, and their removal is an important antioxidant defense mechanism [50]. Hydroxyl radicals can be generated through the Fenton reaction in cells and cause aging and tissue damage [51]. The hydroxyl radical scavenging activities increased as the concentrations of PPP increased; all scavenging rates were higher than those of ascorbic acid. When the PPP concentration reached 5.00 mg/mL, the scavenging rate peaked at 68.81% (Figure 4c), which was higher than that of ascorbic acid, and the EC_50_ value (2.87 mg/mL) of PPP was lower than that of *Pleurotus citrinopileatus* (3.62 mg/mL, Table 3); this also showed that the hydroxyl radical scavenging activity of PPP was stronger than that of *Pleurotus citrinopileatus*.

#### 3.5.4. FRAP Assay

Reducing power is generally related to the presence of reductones exerting antioxidant action by donating a hydrogen atom to halt a free radical chain reaction [51]. In the FRAP assays, PPP at the concentrations tested (0.025–5.00 mg/mL) had strong reducing abilities. When the PPP concentration reached 5.00 mg/mL, FRAP peaked at 4.16 mmol/L (Figure 4d).

In summary, these results showed that PPP had strong antioxidant activities compared with other oyster mushroom polysaccharides or ascorbic acid (Figure 4, Table 3). The reasons involve the structure–activity relationship of PPP and other aspects, which are listed as follows. Analysis of the structures of the polysaccharides in edible fungi revealed that hydroxyl and carboxyl groups can neutralize free radicals by providing hydrogen atoms, thereby blocking free radical chain reactions and reducing oxidative damage [52]. In living organisms, hydroxyl groups can also form disulfide bonds with tyrosine residues in proteins, protecting them from oxidative damage. And carboxyl groups mainly exert antioxidant effects indirectly by binding with other molecules to form ester compounds. Therefore, PPP may effectively remove free radicals in the body and reduce oxidative stress, and it may prevent and treat diseases related to oxidative stress. Hence, PPP could be used as a health food additive to provide many health benefits, such as enhancing immunity and reducing fatigue and aging [32,53,54].

The bioactive and antioxidant components of oyster mushrooms (including *P. pulmonarius*) mainly include two major categories. The first major category is high-molecular-weight compounds such as polysaccharides, glycoproteins, etc.; the second major category is low-molecular-weight compounds like polyphenols, flavonoids, catechols, etc. [7,15]. The low-molecular-weight compounds in *P. pulmonarius* have already been proven to possess strong antioxidant activities (DPPH and ABTS radical scavenging activities, FRAP, etc.), and the contents of total phenols, total flavonoids, and ascorbic acids in different species of *P. pulmonarius* ranged from 4.31 to 19.07 mg/g, 1.76 to 7.79 mg/g, and 0.13 mg/g, respectively (Table 4); the chemical profiles were also determined [10,11,12,55,56]. Therefore, the antioxidant activities, contents, and chemical profiles of low-molecular-weight compounds in *P. pulmonarius* were not included in this study; they are one of the future research directions for this wild *P. pulmonarius* strain.

### 3.6. Cytotoxicity of PPP on Liver and Breast Cancer Cells

Hepatocellular carcinomas and breast cancer pose serious threats to human health [13,17]. The antioxidant activities of many polysaccharides in edible fungi have also been suggested to contribute to their anticancer effects [13,15]. An important attribute of an efficient anticancer drug is cancer selectivity in its cytotoxic effect. Therefore, the cytotoxic effect and anticancer potentials of *P. pulmonarius* polysaccharides on hepatocellular carcinomas and breast cancer were explored based on their antioxidant activities. Cell-based experiments can be used to assess bioactive substances’ mechanisms of action under conditions mimicking the complex environment in organisms; these experiments are also quicker compared to animal experiments [16]. HepG2 cells are used in a wide range of applications, from oncogenesis to the cytotoxicity of substances to the liver. MDA-MB-468 cells are a triple-negative breast cancer cell line with high metastatic potential [57]. Hence, the cytotoxic effects of PPP on HepG2 and MDA-MB-468 cells (typical cancer cells of hepatocellular carcinomas and breast cancer, respectively) were explored by using the MTT assay in this study. The MTT assay, also known as the MTT colorimetric assay, is a method for detecting cell survival and growth. The detection principle is that the succinate dehydrogenase in the mitochondria of living cells can reduce the exogenous MTT to water-insoluble blue-violet crystalline filth (formazan) and deposit it in the cells, while dead cells do not have this function [58]. Dimethyl sulfoxide (DMSO) can dissolve the formazan, and its light absorption value was measured at 490 nm by enzyme immunoassay, which can indirectly reflect the number of living cells [58]. Cisplatin, a widely utilized chemotherapy agent, is a crucial antineoplastic agent in the treatment of various cancer types. As a reference drug, cisplatin was employed to compare and assess the cytotoxic efficacies of PPP [59].

**Table 3:** The EC_50_ values (antioxidant activities) and IC_50_ values (cytotoxic effects).

Samples	EC_50_ Values (mg/mL)			IC_50_ Values (mg/mL)		References
	ABTS Radical Scavenging Activity	DPPH Radical Scavenging Activity	Hydroxyl Radical Scavenging Activity	HepG2 Cell	MDA-MB-468 Cell	
*P. pulmonarius* polysaccharides	0.051	3.322	2.87	1.501	2.183	Present study
*Pleurotus citrinopileatus* polysaccharides	0.063	1.212	3.624	1.690	1.762	[16]
Ascorbic acid	0.00066	0.017	1.132			
Cisplatin				0.00122	0.00287	[59]

**Table 4:** The contents and chemical profiles of low-molecular-weight compounds of *P. pulmonarius.*

	The Contents		The Chemical Profiles	References
Total Phenolic Content (mg/g)	Total Flavonoids (mg/g)	Ascorbic Acid (mg/g)		
5.79 (mg GAE/g)	1.76 (mg QE/g)	0.13		[11]
5.94–11.07 (mg GAE/g)	-	-	phenylvaleric acid, malic acid, and hydroxycinnamic acid derivatives, ascorbic acid, pyroglutamic acid, gallic acid, pyroglutamic acid derivative, cinnamic acid and its hydroxycinnamic acid derivatives, hydroxybenzoic acid derivatives	[10]
4.31 (mg GAE/g)	7.79 (mg QE/g)	-		[55]
6.32–19.07 (mg GAE/g)	-	-	136 chemical constituents were detected	[12]
17.31–21.15 (mg CE/g)	-	-		[56]

-: Not detected; GAE, gallic acid equivalents; CE, catechin equivalents; QE, quercetin equivalents.

**Figure 5:**
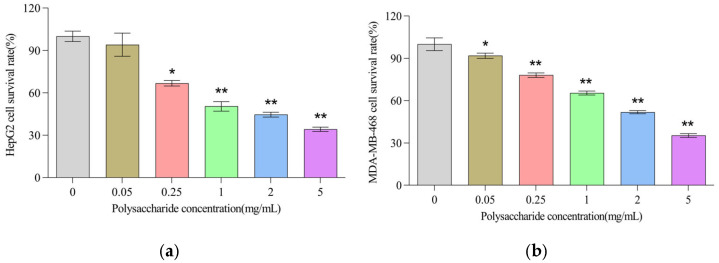
Effects of *P. pulmonarius* polysaccharides (PPPs) on cell viability. (**a**) HepG2 cell viability. (**b**) MDA-MB-468 cell viability. * *p* < 0.05, ** *p* < 0.01 vs. control group (PPP concentration of 0 mg/mL).

As shown in Figure 5a,b, PPP concentration-dependently decreased the viability of both cancer cells at 0.05 (or 0.25)–5.00 mg/mL (*p* < 0.05 or *p* < 0.01). The half inhibitory concentration values (IC_50_) showed that the effect was more pronounced in the HepG2 cells (1.501 mg/mL) than in the MDA-MB-468 cells (2.183 mg/mL) (Table 4). *Pleurotus citrinopileatus* polysaccharides were also found to have cytotoxic effects on HepG2 and MDA-MB-468 cells, but the cytotoxic effect of PPP on HepG2 was stronger than that of *Pleurotus citrinopileatus*, though it was weaker than that of cisplatin [16,59]. The reason why IC_50_ values of the cytotoxic effects or the EC_50_ values of the antioxidant activities of PPP were much higher than those of positive controls (cisplatin or ascorbic acid) are possibly as follows: on one hand, PPP polysaccharides are a mixture, while the positive controls (cisplatin or ascorbic acid) are high-purity compounds; on the other hand, the molecular weight of PPP is much higher than that of these positive controls, and this results in a excessive mass concentration of PPP.

The uncontrolled proliferation of cancer cells is the most basic biological feature of tumors, which is caused by the frequently occurring cell cycle dysregulation during tumorigenesis; blocking cell cycle progression may be an effective strategy for eliminating cancer cells, which is also the main mechanism of several antitumor agents underlying the inhibition of tumor cell proliferation [57]. The results suggested an antigrowth potential of *P. pulmonarius* on liver cancer and breast cancer cells in this study. The possible antigrowth mechanism in liver cancer cells was that PPP caused an accumulation of liver cancer cells in the G2 phase; the proapoptotic effect of PPP on these cells may be caspase-mediated [13]. The possible antigrowth mechanism in breast cancer cells was that PPP might trigger breast cancer cell cycle arrest also at the G2 phase and significantly downregulate the expression of CDK1 and cyclin B1; the activation of CDK1/cyclin B1 complex is a pivotal regulator to promote the cell cycle from G2 to M phase [57].

Various studies have shown that extracts derived from oyster mushroom are rich in polysaccharides like β-glucan and other macromolecules that have an anti-proliferative effect against cancer cell lines, without harming the normal cells [14], and Lavi et al. found that PPP is a mixture of both α- and β-glucans. Therefore, this may be another possible reason for the toxicity of PPP to tumor cells. It was proved that the polysaccharides of oyster mushroom have cytotoxicity and can cause apoptosis of breast cancer cells [14,60]; this also provided indirect evidence for the cytotoxicity of PPP toxicity to breast cancer cells. In addition, it was found that PPP mainly consisted of glucose and galactose, as well as mannose, rhamnose, arabinose, and ribose [60].

Edible fungi including *P. pulmonarius* have been used as a dietary food source for thousands of years, and they are key sources of nutrients for humans; it can be concluded that the polysaccharides in *P. pulmonarius* have no cytotoxic effects on normal human cells. In addition, Pieniadz et al. found that PPP extracted by boiling water did not lead to significant changes in normal cell viability, and PPP extracted by cold water even increased the viability of normal cells by about 20% compared to the untreated control [18]. PPPs were also demonstrated to have selective cytotoxicity to cancer cells [13]. Therefore, the cytotoxic effects on normal human cells were not analyzed in this study.

This study showed that PPPs have cytotoxic effects on liver and breast cancer cells, which provides a new direction and material basis for further functional food and drug development.

## 4. Conclusions

A wild mycorrhizal fungal substrate (strain no. X2118) collected from the Tibet Plateau of China was identified as a novel white *P. pulmonarius* according to morphological and molecular characterization of fruiting bodies. The flesh is tough and white to milky white, a bit different from the non-white appearances of traditional *P. pulmonarius*. Starch was an appropriate carbon source, beef powder was an appropriate nitrogen source, the appropriate temperature was 30 °C, and pH 6 was an appropriate pH value. *P. pulmonarius* had a short growth cycle and high yield after it was domesticated. Nutritional analysis revealed that the fruiting bodies were important sources of carbohydrates, fibers, proteins, essential amino acids, and minerals. PPP from the fruiting bodies had strong antioxidant activities and cytotoxic effects on cancer cells of HepG2 and MDA-MB-468 cells. This study provides theoretical support for expanding the international edible mushroom germplasm resource, adapting and scaling up the cultivation of *P. pulmonarius* from high-altitude Tibet to low-altitude regions. The next research directions for this wild *P. pulmonarius* strain will primarily focus on the following areas: (1) the antioxidant activities, contents, and chemical profiles of low-molecular-weight compounds in this wild *P. pulmonarius* strain; (2) other activities of PPP and the corresponding mechanisms; and (3) the research and development of *P. pulmonarius* functional foods.

## Data Availability

The original contributions presented in the study are included in the article, further inquiries can be directed to the corresponding author.
